# Involvement of Actin Cytoskeletal Components in Breast Cancer Cell Fusion with Human Mesenchymal Stroma/Stem-Like Cells

**DOI:** 10.3390/ijms20040876

**Published:** 2019-02-18

**Authors:** Catharina Melzer, Juliane von der Ohe, Ralf Hass

**Affiliations:** Biochemistry and Tumor Biology Lab, Department of Obstetrics and Gynecology, Hannover Medical School, D-30625 Hannover, Germany; Melzer.Catharina@mh-hannover.de (C.M.); Ohe.Juliane.von.der@mh-hannover.de (J.v.d.O.)

**Keywords:** hybrid cells, fusion, mesenchymal stem cells, breast cancer, cytoskeleton, tumor microenvironment

## Abstract

Cell fusion as a rare event was observed following the co-culture of human MDA-MB-231^cherry^ breast cancer cells or benign neoplastic MCF10A^cherry^ breast epithelial cells together with different mesenchymal stroma/stem-like cells (MSC^GFP^) cultures, respectively, resulting in the generation of double-fluorescing hybrid cells. Analysis of potential molecular mechanisms for the formation of cancer hybrid cells revealed cytoskeletal components, including F-actin. Thus, a sub-lethal concentration of cytochalasin D, which blocks elongation of actin filaments, was able to significantly reduce cancer hybrid cell formation. Simultaneously, cell cycle progression of the different co-cultures remained unaffected following treatment with cytochalasin D, indicating continued proliferation. Moreover, exposure to 50 nM cytochalasin D revealed little if any effect on the expression of various integrins and cell adhesion molecules in the different co-cultures. However, LC-MS proteome analysis of the different control co-cultures compared to corresponding cytochalasin-treated co-cultures demonstrated predominant differences in the expression of actin-associated cytoskeletal proteins. In addition, the requirement of structured actin to provide an appropriate cytoskeletal network for enabling subsequent fusion processes was also substantiated by the actin filament disrupting latrunculin B, which inhibits the fusion process between the breast cancer populations and mesenchymal stroma/stem-like cells (MSC). Together, these findings suggest an important role of distinct actin structures and associated cytoskeletal components during cell fusion and the formation of breast cancer hybrid cells.

## 1. Introduction

Cancer cell fusion can generate new populations of cancer hybrid cells, either by homotypic (autofusion—the combination of cells from the same population) or heterotypic (heterofusion—the hybrid formation of different cell types) processes [1,2,3]. These rare processes are accompanied by a recombination of genomic parts from both parental donors, in a nuclear hetero-to-synkaryon transition during subsequent cell division [4]. The transfer or exchange of genomic compounds have been detected by various studies, providing evidence for in vivo hybrid cell formation in several carcinomas [2,5,6,7]. Thus, cancer cell fusion enables RNA and DNA transfer to non-cancer cells, such as mesenchymal stroma/stem-like cells (MSC) and vice versa, contributing to massive genomic alterations and clonal diversity [8,9,10,11,12]. Consequently, this genetic change of a hybrid cell alters its fundamental biological properties and cancer cell fate. In addition, cell fusion can generate aneuploidy, chromosomal instability, mutations, and DNA damage, which are accompanied by multiple genetic aberrations and potentially neoplastic development [13]. An alternative generation of hybrid cells can occur by engulfment of a target cell via entosis; however, this includes degradation of the target cell genome [4]. Moreover, cannibalism may also represent a mechanism for hybrid cell generation whereby cancer cells can enter dormancy after cannibalizing MSC [14].

Previous work has demonstrated that cell fusion in vitro requires a hypoxic environment, together with an optimized pH and proper alignment of certain glycoproteins, as well as a permissive lipid composition of the involved parts of the cell membranes to facilitate the initiation of a fusion event [15]. Indeed, previous work has demonstrated that hypoxia-induced apoptosis stimulates fusion between breast cancer cells and MSC, displaying enhanced migratory capacity of the newly formed hybrids [16]. Apart from the rare, pathophysiological, so-called “accidental cell fusion” for aberrant spontaneous hybrid cell formation, physiological homo- and heterofusion during normal development of certain tissues include, among other things, repetitive myoblast fusion to form to multi-nucleated myocytes in muscle fibers [17], trophoblast fusion to form syncytiotrophoblasts in developing placenta, and the fusion of egg and sperm to develop a new organism [18].

Although the molecular triggers and precise mechanism of hybrid cell formation remain unclear to date [19], previous work has identified distinct factors displaying fusogenic properties. These include truncated mammalian genes originating from human endogenous retrovirus W (HERV-W) retroviral envelope genes and encoding the proteins syncytin-1 and -2, predominantly expressed in placental syncytiotrophoblasts [20,21]. Indeed, while cell fusion can be also detected in breast cancers and others [22,23], endothelial ASCT2 (alanine, serine, and cysteine selective transporter-2) can function as a syncytin receptor to mediate the heterofusion of breast cancer cells with endothelial cells [24,25]. Moreover, other work has demonstrated that molecular signals triggering the fusion of different breast cancer cells with MSC include pro-inflammatory cytokines like TNF-α and signaling by TNF receptor-1 and -2, via the associated downstream factor tumor necrosis factor receptor type 1-associated death domain protein (TRADD) [26].

While the actin-based cytoskeleton was previously suggested to be involved, e.g., in myoblast fusion [27], the present work demonstrates fusion of different cultures of MSC with highly malignant breast cancer cells, as well as benign neoplastic breast epithelial cells, to generate new hybrid cell populations. Following investigations of potential molecular factors involved, we provide evidence for a predominant involvement of actin fibers in this cancer cell fusion process.

## 2. Results

**Figure 1:**
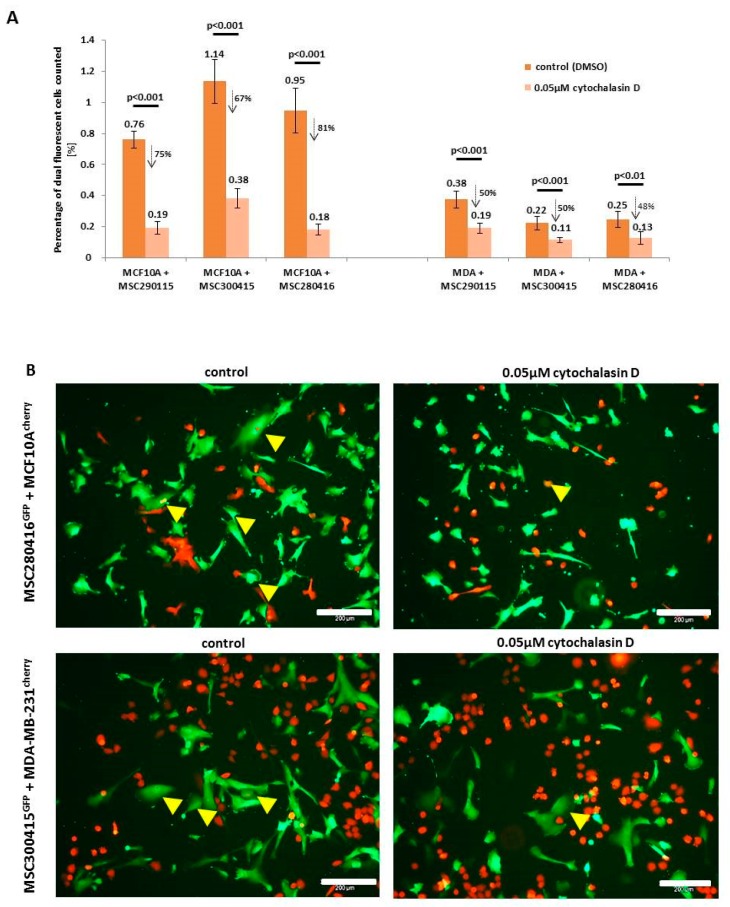
Effect of actin polymerization inhibitor on hybrid cell formation. (**A**) Initially, 6000 cells per well (50:50) of co-cultures between MSC^GFP^ from three different donors (MSC290115, MSC300415, and MSC280416), as well as MCF10A^cherry^ or MDA-MB-231^cherry^ were started overnight to allow attachment. After 24 h, co-cultures were treated with either 0.05 µM cytochalasin D or a solvent concentration of DMSO, and the percentage of dual fluorescent cells was determined after 24 h of treatment. Data represent the mean ± SD from *n* = 6, and significances were calculated using a Student′s *t*-test. (**B**) Fluorescence microscopy of co-cultures between MSC280416^GFP^ and benign MCF10A^cherry^ neoplastic breast epithelial cells (left panel) and fluorescence microscopy of co-cultures between MSC300415^GFP^ and malignant MDA-MB-231^cherry^ breast cancer cells (right panel) are exemplarily demonstrated in control co-cultures (stimulated with solvent concentration of DMSO), and in the presence of 0.05 µM cytochalasin D for 24 h. Scale bars: 200 µm.

While heterofusion between MSC and neoplastic breast cells represents a rare event that occurs in 0.2% to about 1% of the co-cultured population, treatment with actin polymerization inhibitor cytochalasin D significantly reduces the rate of hybrid cell formation (Figure 1A). Experiments were performed with MSC from three different donors, and hybrid cell formation was quantified for dual fluorescent cells, after cytochalasin D stimulation in co-cultures with benign neoplastic MCF10A mammary epithelial cells (Figure 1A, left panel) and malignant triple negative MDA-MB-231 breast cancer cells (Figure 1A, right panel). Cell fusion declined in the three cytochalasin D-treated MSC-MCF10A co-cultures by about 74.2% (MSC290115/MCF10A by 75%, MSC300415/MCF10A by 67%, and MSC280416/MCF10A by 81%) (Figure 1A, left panel). Likewise, cytochalasin D reduced the amount of fused hybrid cells in co-cultures of MDA-MB-231 cells together with the three different MSC populations by about 50% (MSC290115/MDA-MB-231 by 50%, MSC300415/MDA-MB-231 by 50%, and MSC280416/MDA-MB-231 by 48%) (Figure 1A, right panel). Moreover, the effects of cytochalasin D on hybrid cell formation and on cell morphology are represented by exemplarily chosen fluorescence microscopic images from one out of each of the three co-cultures tested. Dual fluorescent cells are highlighted with yellow arrowheads, and reflect the decreased amount of hybrid cells during cytochalasin D treatment (Figure 1B).

**Figure 2:**
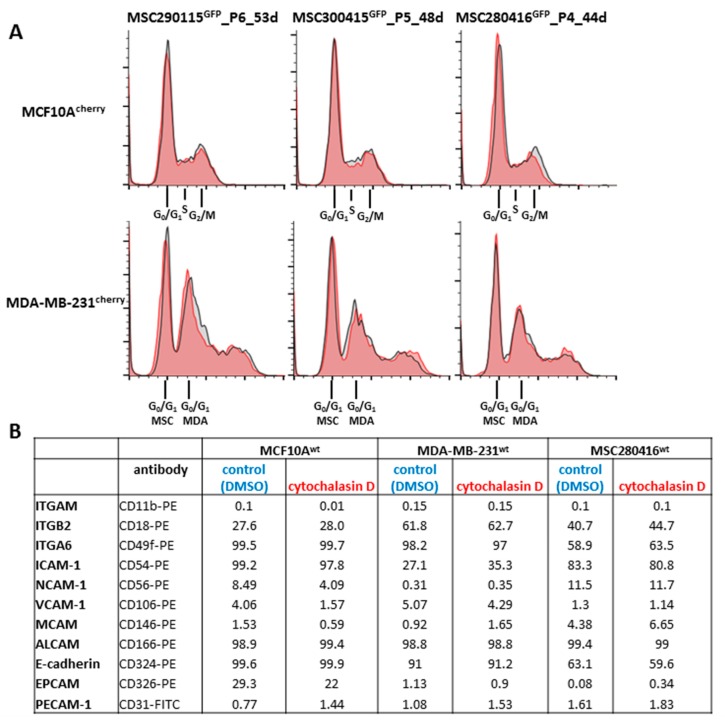
Cell cycle analysis and surface marker expression during cytochalasin D stimulation. (**A**) Cell cycle analysis of the indicated co-cultures was performed by DNA labeling with propidium-iodide and analyzed via flow cytometry. The grey histograms represent control co-cultures in solvent dilution (DMSO), and the red histograms demonstrate exposure to 0.05 µM cytochalasin D in DMSO for 24 h. (**B**) Cell surface marker expression analysis of different cell adhesion molecules in MCF10A^wt^, MDA-MB-231^wt^, and MSC280416^wt^ cell populations was examined by flow cytometry, and the percentage of appropriate expression is documented in control cells after the appropriate solvent dilution (DMSO), as compared to treatment with 0.05 µM cytochalasin D in DMSO for 24 h, respectively.

Additional effects of cytochalasin D were analyzed on cell cycle traverse and adhesion molecule expression (Figure 2). The cell cycle progression of co-cultures from the three different MSC^GFP^ populations, together with either MCF10A^cherry^ (Figure 2A, upper panel, grey histograms) or with MDA-MB-231^cherry^ (Figure 2A, lower panel, grey histograms), demonstrated little if any difference compared to a 24 h treatment with 0.05 µM cytochalasin D of the corresponding MSC^GFP^/MCF10A^cherry^ (Figure 2A, upper panel, red histograms) or MSC^GFP^/MDA-MB-231^cherry^ co-cultures (Figure 2A, lower panel, red histograms), respectively. These findings suggest a limited involvement of cell cycle progression in cell fusion events between MSC and benign or malignant breast cancer cell populations.

Different cell adhesion molecules that contribute to intercellular communication processes were analyzed by flow cytometry in steady-state MCF10A^wt^, MDA-MB-231^wt^, and MSC280416^wt^ mono-cultures (DMSO solvent control), after 24 h exposure to 0.05 µM cytochalasin D. No significant differences in cell adhesion molecule expression were detectable in the three control versus cytochalasin D-treated cell cultures (Figure 2B).

**Figure 3:**
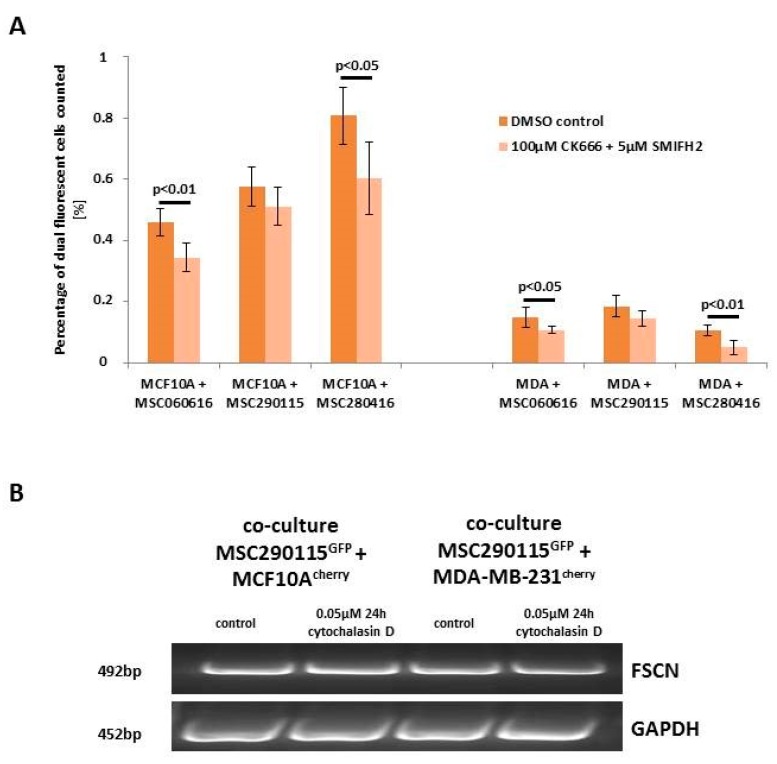
Quantification of hybrid cell formation during treatment with CK666 and SMIFH2, as well as PCR analysis of fascin. (**A**) Dual fluorescent cell formation was evaluated in co-cultures (MSC^GFP^ from three different donors with breast cancer cell populations MCF10A^cherry^ and MDA-MB-231^cherry^) after 24 h treatment with 100 µM actin-related protein (Arp2/3) complex inhibitor CK666, and 5 µM cell-permeable inhibitor of formin homology 2 (FH2) domains (SMIFH2). Data represent the mean ± SD (*n* = 6), and significances were calculated by Student´s *t*-test. (**B**) Co-cultures of MSC290115^GFP^ with MCF10A^cherry^ and MDA-MB-231^cherry^, respectively, were stimulated for 24 h with 0.05 µM cytochalasin D, and expression of fascin was examined by PCR analysis. Transcript levels of GAPDH served as a control.

Next, we examined the influence of cytosolic proteins associated with actin polymerization and branching. A combination of two drugs that inhibit lamellipodia and filopodia formation was tested in co-cultures of three different MSC^GFP^ with MCF10A^cherry^ or MDA-MB-231^cherry^, respectively (Figure 3A). CK666 represents an inhibitor of the Arp2/3 complex, producing branched filaments, and SMIFH2 inhibits formin, which produces unbranched filaments [28]. The combined treatment of these inhibitors was examined under six different conditions for each co-culture, and resulted in a significantly reduced hybrid cell formation in four out of the six differently treated co-cultures when compared to the corresponding control co-cultures (Figure 3A).

While these data suggest the involvement of lamellipodia or filopodia formation in cell fusion mechanisms, we validated the expression level of fascin, a major F-actin bundling protein in co-cultures treated with and without cytochalasin D. However, little if any difference in fascin expression levels was detectable by PCR analysis, suggesting steric effects during formation of filopodia, which are known to be caused by cytochalasin D (Figure 3B) [29].

**Figure 4:**
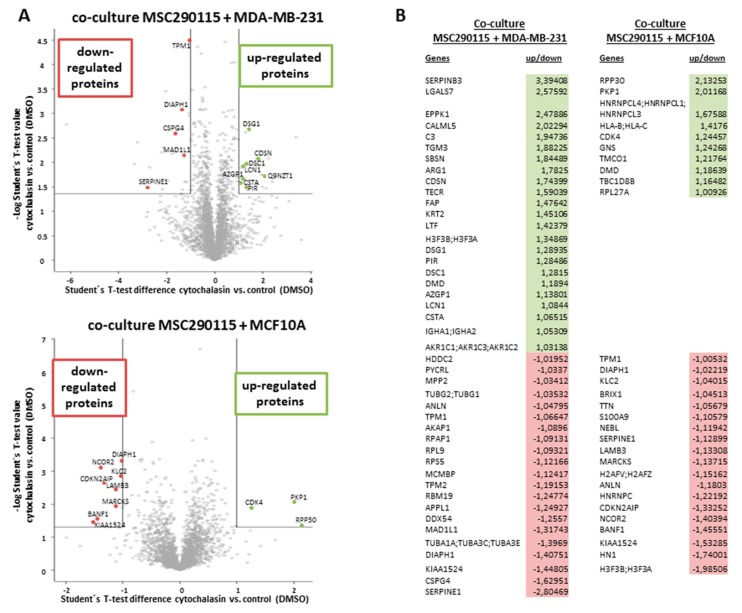
Mass spectrometry (MS) analysis of cytochalasin D-stimulated co-cultures. (**A**) Proteome analysis by mass spectrometry for up-regulated (green) and down-regulated (red) proteins in co-cultures between MSC290115^GFP^ and MDA-MB-231^cherry^ breast cancer cells (upper histogram), as well as co-cultures of MSC290115^GFP^ with the benign neoplastic MCF10A^cherry^ population (lower histogram) after stimulation in the absence (control co-cultures) or presence of 0.05 µM cytochalasin D for 24 h, is demonstrated, together with selected protein spots in the corresponding volcano blots. (**B**) Significantly up- and down-regulated proteins are summarized compared to the fold induction/reduction. Positive values (green) represent up-regulated proteins in cytochalasin D-treated co-cultures versus control co-cultures, whereas down-regulated proteins (negative values) are demonstrated in red, following cytochalasin D exposure compared to control co-cultures.

Consequently, a whole proteomic analysis was examined with co-cultures of MSC290115^GFP^ and MDA-MB-231^cherry^ (Figure 4A, upper histogram), as well as co-cultures of MSC290115^GFP^ together with MCF10A^cherry^ (Figure 4A, lower histogram) after 24 h of stimulation with 0.05 µM cytochalasin D, which were compared to DMSO solvent control co-cultures, respectively. The results are presented in a volcano plot demonstrating up-regulated (green area and selected spots) and down-regulated proteins (red area and selected spots) (Figure 4A). All significantly altered proteins are summarized in a table (Figure 4B). Thus, in cytochalasin D-treated MSC290115/MDA-MB-231 co-cultures, 23 proteins were up-regulated and 21 proteins were down-regulated versus the control co-cultures (Figure 4B, left panel). Moreover, inhibition of actin polymerization in co-cultures of the same MSC290115 with MCF10A cells revealed 10 up-modulated and 19 down-modulated proteins (Figure 4B, right panel). Of interest, a variety of actin-associated proteins, including anilin, tropomyosin 1 and 2, tubulin, and DIAPH1 (diaphanous-related formin 1) were downregulated, and DMD (dystrophin) was upregulated in the presence of cytochalasin D.

**Figure 5:**
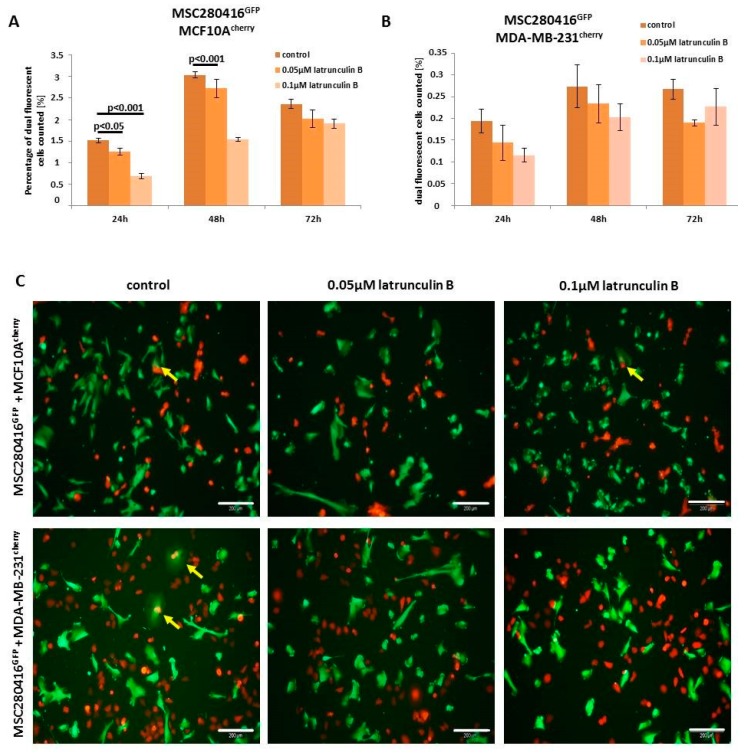
Fusion cell quantification in co-cultures treated with latrunculin B. (**A**,**B**) Co-cultures of MSC280416^GFP^ with MCF10A^cherry^ or MDA-MB-231^cherry^ were treated with 0.05 µM and 0.1 µM latrunculin B for 24 h, and hybrid cell formation was calculated by cell counting. Significance (*p*) was calculated by the mean ± SD from triplicates (*n* = 3) using ANOVA followed by Dunnett´s multiple comparisons test. (**C**) Fluorescent microscopic images of co-cultures treated with 0.05 µM and 0.1 µM latrunculin B were compared to control co-cultures. Bars represent 200 µm.

Besides the inhibition of actin polymerization by the binding of cytochalasin D to the barbed end of actin filaments, latrunculin B also represents a potent inhibitor of actin polymerization by binding to actin monomers [30]. Treatment of MSC280416^GFP^/MCF10A^cherry^ co-cultures with different concentrations of latrunculin B for up to 72 h resulted in significantly decreased cell fusion (Figure 5A). Similar data with reduced hybrid cell formations were observed in MSC280416^GFP^/MDA-MB-231^cherry^ co-cultures after latrunculin B exposure (Figure 5B). An altered morphology and reduction in the number of fusion cells is demonstrated by fluorescence microscopy images of control co-cultures compared to latrunculin B-treated co-cultures, whereby the appearance of dual fluorescent cells is highlighted with yellow arrows (Figure 5C).

## 3. Discussion

Several multi-modal direct or indirect interaction mechanisms can occur between cancer cells and MSC, which last for several hours or even days [31,32,33,34]. One of these direct interactions is represented by cell fusion, which can be observed in human MSC together with human breast cancer cells within less than five minutes [26]. The known fusogenic proteins syncytin-1 and syncytin-2, together with the corresponding receptors ASCT2 and MFSD2A for syncytiotrophoblast fusion, are also linked to tumorigenic processes, whereby downregulation of syncytin-1 inhibits cell fusion between breast cancer cells and endothelial cells [35]. Other studies have demonstrated additional selective and more cell type-specific molecular fusion signals, such as TNF receptor activation during the spontaneous cell fusion of MSC with neoplastic breast epithelial cells. Moreover, a ten-fold lower generation of hybrid cells by autofusion compared to corresponding heterofusion indicates a fusion-permissive environment by an assembly of distinct molecular structures in different cellular fusion partners, rather than during homotypic hybrid cell formation [26]. Thus, the present findings of fusion inhibition by cytochalasin D suggests the involvement of the actin cytoskeleton. Supportive data are presented in a mouse model demonstrating the importance of the Rho–ROCK–actin/myosin signaling cascade for cell fusion and entosis in mouse embryonic stem cells [4]. Moreover, previous work has demonstrated a substantial inhibition of CD90 and CD105 membrane protein transfer by cytochalasin D during the interaction between MSC and breast cancer or ovarian cancer cells, respectively [36]. This intercellular protein traffic via nanotubes requires actin microfilaments to perform traction and contraction forces, which can be blocked by cytochalasin D-mediated inhibition of actin polymerization. Likewise, an exchange of mitochondria via nanotubes-containing actin microfilaments between MSC and vascular smooth muscle cells can be abolished by cytochalasin D [37]. Cell cycle progression of the different co-cultures remains unaltered during cytochalasin D exposure, suggesting more specific effects on fusion inhibition. A predominant involvement of actin and associated cytoskeletal components is also supported by findings that treatment with cytochalasin D exhibits little if any detectable effects on the expression of integrins and various cell adhesion molecules, which also play an important role during intercellular communication of breast cancer cells and MSC.

Interference with the formation of lamellipodia via Arp2/3, and filopodia via formin by CK666 and SMIFH2, respectively, demonstrates a significant reduction of cancer hybrid cell formation with different MSC co-cultures, also substantiating the role of actin and associated cytoskeletal components in these fusion processes. This is further evidenced by the comparative proteome analysis of different breast cancer co-cultures during cytochalasin D exposure, which predominantly reveals altered expression of actin-associated cytoskeletal components. Finally, latrunculin B significantly down-modulated fusion events in co-cultures of breast cancer cells with MSC. Latrunculins belong to a family of macrolide-structured toxins, and latrunculin B predominantly impairs the building of an actin cytoskeleton by binding to monomeric G-actin, preventing complex formation with ATP, which is required for the polymerization of filamentous F-actin [29].

Together, these findings suggest a substantial role of proper actin polymerization and associated cytoskeletal protein alignment to enable a fusion-permissive microenvironment of the fusogenic cellular partners. 

## 4. Materials and Methods 

### 4.1. Cell Culture

#### 4.1.1. Breast Cancer Cells

Human breast carcinoma cell lines MDA-MB-231 and MCF10A were commercially obtained from American Type Culture Collection. The triple-negative breast cancer cell line MDA-MB-231 was grown in Leibovitz´s L-15-medium (Life Technologies, Darmstadt, Germany), supplemented with 10% fetal calf serum (FCS), 100 U/mL penicillin, 100 µg/mL streptomycin, and 2 mM L-glutamine (Sigma Chemie GmbH, Taufkirchen, Germany). The benign breast carcinoma cell line MCF10A was cultured in phenol red-free mammary epithelial cell basal medium (MECBM) with an appropriate growth medium supplement mix (Promocell, Heidelberg, Germany).

#### 4.1.2. Mesenchymal Stroma/Stem Cells

The usage of primary human MSC was approved by the Ethics Committee of Hannover Medical School, Project #443 on February 26th, 2009, and informed written consent was obtained from each patient. MSC were isolated from umbilical cord tissue explant cultures, as described previously [38], and cultured in αMEM (Sigma Chemie GmbH, Steinheim, Germany) supplemented with 10% allogeneic human AB-serum, 100 U/mL penicillin, 100 µg/mL streptomycin, and 2 mM L-glutamine (Sigma Chemie GmbH). For the experiments, MSC cultures were used from four different donors: MSC290115, MSC300415, MSC280416, and MSC060616.

All cells were tested for mycoplasma by the luminometric MycoAlert Plus mycoplasma detection kit (Lonza Inc., Rockland, ME, USA), according to the manufacturer’s recommendations. Authentication of the human breast carcinoma cell lines was performed by short tandem repeat (STR) fragment analysis, using the GenomeLab human STR primer set (Beckman Coulter Inc., Fullerton, CA, USA).

For discrimination of MSC and breast cancer cell lines in co-culture, cell populations were stably transduced with a third-generation lentiviral SIN vector carrying either the *mCherry* gene (MDA-MB-231^cherry^ or MCF10A^cherry^) or the enhanced green fluorescent protein (*eGFP*) gene (MSC^GFP^). Co-cultures of MSC^GFP^ and MDA-MB-231^cherry^ were cultivated in αMEM, whereas co-cultures of MSC with MCF10A were grown in 60% MECBM and 40% αMEM.

### 4.2. Quantification of Hybrid Cells

The quantification of hybrid cells was performed as previously described [26]. Briefly, co-cultures with 50% GFP-labeled MSC and 50% cherry-labeled breast cancer cell lines were initiated in 24-well plates. At indicated time points, the total number of dual fluorescent cells per well was quantified using a fluorescence microscope (Olympus IX50), with red and green fluorescence filters and an FITC/TRIC dual band filter. After quantifying the amount of dual fluorescent cells per well, the co-cultures were trypsinized, and the total cell number was determined in a hemocytometer to compute the percentage of hybrid cells.

### 4.3. Flow Cytometric Analysis

For flow cytometric analysis, the samples were first blocked with 2% FCS in PBS for 15 min at room temperature. After a PBS washing step, the cells were stained with appropriate PE- or FITC-labeled antibodies at 4 °C for 15 min. The following antibodies were used: mouse monoclonal CD11b-PE (clone 2LPM19C, IgG1, Dako, Agilent, Santa Clara, CA, USA); mouse monoclonal CD18-PE (clone TS1/18, IgG1, Miltenyi Biotech GmbH, Bergisch Gladbach, Germany); rat CD49f (clone GoH3, IgG2a, Miltenyi Biotech GmbH); mouse monoclonal CD54-PE (clone HCD54, IgG1, BioLegend, San Diego, CA, USA); CD56-PE (clone MEM-188, IgG2a, ImmunoTools GmbH, Friesoythe, Germany); mouse monoclonal CD106-PE (clone STA, IgG1, BioLegend); rat CD146-PE (clone ME-09F1, IgG2a, Miltenyi Biotech GmbH); mouse monoclonal CD166-PE (clone REA442, IgG1, Miltenyi Biotech GmbH); mouse monoclonal CD324-PE (clone 67A4, IgG1, Miltenyi Biotech GmbH); CD326-PE (clone HEA-125, Miltenyi); and mouse monoclonal CD31-FITC (clone WM59, IgG1, Dako, Agilent). PE- or FITC-labeled antibodies of the corresponding IgG subclass (Dako, Agilent) served as a control. Thereafter, the cells were washed again with PBS and subsequently analyzed by a flow cytometer using FACSCalibur (BD Biosciences GmbH, Heidelberg, Germany) and FlowJo V10 software.

### 4.4. Cell Cycle Analysis

The cell cycle analysis was performed as described previously [39]. Briefly, 1 × 10^5^ cells were fixed in 70% ice-cold EtOH at 4 °C for 24 h. The fixed cells were then stained with propidium iodide (PI) staining solution containing 12.5 µg/mL PI, 0.5% Triton-X-100, and 100 U/mL DNase-free RNase in PBS for 30 min at room temperature. Thereafter, the samples were measured by flow cytometry (FACScalibur, BD Biosciences GmbH) and analyzed by FlowJo V10 software.

### 4.5. Transcript Analysis by Reverse Transcription Polymerase Chain Reaction

The total cellular RNA was isolated using an RNeasy Mini Kit (Qiagen, Hilden, Germany) according to the manufacturer’s protocol. Briefly, 1 µg RNA was reverse transcribed into cDNA using 500 µM of dNTP, 5 µM of Random Hexan primer, 5 µM of Oligo(dT) primer, 1 U of RiboLockTM RNase Inhibitor, and 5 U of RevertAidTM Reverse Transcriptase in the supplied reaction buffer (all reagents from Thermo Scientific, Schwerte, Germany). The cDNA reaction was performed for 10 min at 25 °C, followed by 1 h at 42 °C, and stopped at 72 °C for 10 min. Then, 2.5 µL of cDNA were amplified by polymerase chain reaction (PCR) with the following specific primers (customized by Eurofins MWG GmbH, Ebersberg, Germany):

- FSCN (sense: 5′-CTG GCT ACA CGC TGG AGT TC-3′; antisense: 5′-CTG AGT CCC CTG CTG TCT CC-3′; amplification product 492 bp)
- GAPDH (sense: 5′-ACC ACA GTC CAT GCC ATC AC-3′; anti-sense: 5′-TCC ACC ACC CTG TTG CTG TA-3′; amplification product 452 bp)

The PCR reaction included 200 nM of each primer, 200 µM of dNTP, and 0.03 U One Taq Hot Start DNA polymerase (New England Biolabs GmbH, Frankfurt am Main, Germany) in the appropriate buffer. The PCR cycling conditions included 30 s at 95 °C, 1 min at 60 °C, and 1 min at 72 °C, with an initial 5 min denaturation step at 95 °C and a final 5 min extension step at 72 °C (35 cycles). Aliquots of 25 µL of PCR products were separated on a 2% agarose gel using the standard GeneRuler 100 bp DNA Ladder (Thermo Scientific). Visualization was performed by GelRed staining (Biotium Inc., Fremont, CA, USA).

### 4.6. Mass Spectrometric Analysis

#### 4.6.1. Sample Preparation for Mass Spectrometry Analysis

Protein from co-cultures in the absence (control) or presence of cytochalasin D was mixed with 5× loading buffer (2.5M Tris-HCl, 40% glycerin, 2.5% SDS, 25mM DTT, 10 µg bromophenol blue) and incubated for 5 min at 95 °C. Proteins were then alkylated by addition of acrylamide up to a concentration of 2%, and incubation at room temperature (RT) for 30 min. SDS-PAGE was performed on 10% gels. After electrophoresis, the proteins were fixed with 40% (*v*/*v*) Ethanol and 10% (*v*/*v*) acetic acid in water for 60 min, and stained with Coomassie Brilliant Blue overnight. Background staining was reduced with 25% (*v*/*v*) methanol in water. Each lane was cut into four pieces, which were further minced to 1 mm³ gel pieces. Subsequent sample processing was performed as described [40]. Briefly, gel pieces were de-stained two times with 200 µL 50% acetonitrile (ACN), 50 mM ammonium bicarbonate (ABC) at 37 °C for 30 min, and then dehydrated with 100% ACN. The solvent was removed in a vacuum centrifuge, and 100 µL 10 ng/µL sequencing grade trypsin (Promega) in 10% ACN and 40 mM ABC were added. Gels were rehydrated in trypsin solution for 1 h on ice and then covered with 10% ACN and 40 mM ABC. Digestion was performed overnight at 37 °C, and was stopped by adding 100 µL of 50% can and 0.1% trifluoroacetic acid (TFA). After incubation at 37 °C for 1 h, the solution was transferred into a fresh sample vial. This step was repeated twice, and extracts were combined and dried in a vacuum centrifuge. Dried peptide extracts were re-dissolved in 30 µL 2% can and 0.1% TFA, with shaking at 800 rpm for 20 min. After centrifugation at 20,000× *g*, aliquots of 12.5 µL each were stored at −20 °C.

#### 4.6.2. LC-MS Analysis

Peptide samples were separated with a nano-flow, ultra-high-pressure liquid chromatography system (RSLC, Thermo Scientific), equipped with a trapping column (3 µm C18 particle, 2 cm length, 75 µm ID (Acclaim PepMap, Thermo Scientific, Braunschweig, Germany) and a 50 cm-long separation column (2 µm C18 particle, 75 µm ID; Acclaim PepMap, Thermo Scientific). Peptide mixtures were injected, enriched, and desalted on the trapping column at a flow rate of 6 µL/min with 0.1% TFA for 5 min. The trapping column was switched online with the separating column, and peptides were eluted with a multi-step binary gradient: a linear gradient of buffer B (80% ACN, 0.1% formic acid) in buffer A (0.1% formic acid) from 4% to 25% in 30 min, 25% to 50% in 10 min, 50% to 90% in 5 min, and 10 min at 90% B. The column was reconditioned to 4% B in 15 min. The flow rate was 250 nL/min, and the column temperature was set to 45 °C. The RSLC system was coupled online, via a Nano Spray Flex Ion Soure II (Thermo Scientific), to an LTQ-Orbitrap Velos mass spectrometer. Metal-coated fused-silica emitters (SilicaTip, 10 µm i.d., New Objectives) and a voltage of 1.3 kV were used for the electrospray. Overview scans were acquired at a resolution of 60 k in a mass range of *m*/*z* 300–1600 in the orbitrap analyzer, and stored in profile mode. The top 10 most intensive ions of charges two or three and a minimum intensity of 2000 counts were selected for CID fragmentation, with a normalized collision energy of 38.0, an activation time of 10 ms, and an activation *Q* of 0.250 in the LTQ. Fragment ion mass spectra were recorded in the LTQ at a normal scan rate, and stored as a centroid *m*/*z* value and intensity pairs. Active exclusion was initialized, so that ions fragmented once were excluded from further fragmentation for 70 s within a mass window of 10 ppm of the specific *m*/*z* value.

Raw data were processed using Proteome Discoverer (Thermo Scientific), as well as human and virus uniprot/swissprot databases containing common contaminants. The stated proteins were identified by a false discovery rate of 0.01 at the protein and peptide level, and were quantified by extracted ion chromatograms of all peptides.

## 5. Conclusions

While the present work confirms previous findings of hybrid formation between benign neoplastic breast epithelial cells and different MSC cultures, as well as fusion between malignant breast cancer cells and MSC populations, evidence is provided for an important role of actin filament structures in the fusion process. Thus, cytochalasin D and latrunculin B, which both block actin polymerization via different mechanisms, were capable of selectively inhibiting cell fusion, while no significant effects were observed in other cellular pathways, including cell cycle progression or the expression of various cell adhesion molecules. Moreover, the identification of a panel of actin-associated proteins by comparative proteome analysis further substantiates a predominant involvement of actin cytoskeletal components to provide a fusion-permissive environment. Although a detailed molecular interplay for the regulation of cancer cell fusion processes remains unclear, further evaluation may also unravel the heterogenic properties of resulting cancer hybrid cells, displaying enhanced or reduced tumorigenic and metastatic potential.

## Abbreviations

ABC	ammonium bicarbonate
ACN	acetonitrile
ASCT2	alanine, serine and cysteine selective transporter-2
DIAPH1	diaphanous related formin 1
DMD	dystrophin
eGFP	enhanced green fluorescent protein
FACS	fluorescence-activated cell sorting
FCS	fetal calf serum
MFSD2A	Major facilitator superfamily domain-containing protein 2
MSC	mesenchymal stroma/stem-like cells
PI	propidium-iodide
RT-PCR	reverse transcription polymerase chain reaction
STR	short tandem repeat
TFA	trifluoroacetic acid
